# Nutrition Intervention and Cardiovascular Disease

**DOI:** 10.3390/nu14071435

**Published:** 2022-03-30

**Authors:** Maria Pia Adorni, Nicola Ferri

**Affiliations:** 1 Unit of Neurosciences, Department of Medicine and Surgery, University of Parma, 43125 Parma, Italy; mariapia.adorni@unipr.it; 2 Department of Medicine, University of Padua, 35128 Padua, Italy

Dietary factors influence the development of cardiovascular diseases (CVD) either directly or through their action on traditional risk factors, such as plasma lipids, blood pressure, inflammation, or glucose levels. Many discordant results have been observed due to both methodological problems (particularly inadequate sample sizes or short study durations) and the difficulties of evaluating the impact of a single dietary factor independent of any other changes in the diet. To overcome, at least in part, these problems, in recent years, nutrition research has focused on the relationship between atherosclerotic CVD on the one hand, and foods and dietary patterns, rather than single nutrients, on the other.

The Special Issue “Nutrition Intervention and Cardiovascular Disease” is intended to provide the most recent clinical and preclinical evidence on nutritional intervention for preventing atherosclerotic CVD or modifying risk factors (Figure 1). In this respect, as summarized in the Figure 1, it has been reported that intake of functional foods or dietary components may exert a positive cardiometabolic effect by improving insulin sensitivity in obese patients or variables associated to CV risk in patients with multiple sclerosis. In addition, differential positive effects of dietary fatty acids, sterols and liposoluble vitamins have been documented on reverse cholesterol transport (RCT), the atheroprotective process inversely correlated with CV risk. Still inconclusive data, however, have been obtained on the effect of nutraceuticals such as magnesium, zinc, iron, vitamin K, and phytate on the progression of vascular calcification, a process associated to an increased risk of CVD.

In detail, this Issue includes two clinical research articles describing a randomized, double-blind controlled, crossover trial on obese patients with elevated low-density lipoprotein cholesterol (LDL-C) levels, and a randomized control pilot study in patients with Multiple Sclerosis. The results of the first trial show a significant improvement of insulin resistance, lipid particle profiles, and serum plasminogen activator inhibitor-1 (PAI-1) in response to consumption of strawberries at two-and-a-half servings for four weeks (32 g powder/day) [1]. This is a very well conducted study that further confirms the health benefits of berry fruits, especially blueberries, cranberries, and strawberries, for their high polyphenol and fiber content. Strawberry supplementation has already demonstrated to decrease total and LDL-C, to improve the antioxidant status and to reduce lipid peroxidation and inflammatory response in patients with T2 diabetes; the study presented in this Special Issue reported an additional positive cardiometabolic effect by improving insulin sensitivity in a nonacute setting. These actions are most likely related to the presence of approximately 960 mg of polyphenols in the daily dose of strawberry powder utilized.

The second clinical trial studied the effect on CV risk of a specific polyphenol, such as the epigallocatechin gallate (EGCG), together with 60 mL of coconut oil, as a source of ketone bodies [2]. The study last for 4 months and was conducted in patients affected by multiple sclerosis, a chronic and degenerative disease characterized by anthropometric changes related to CV risk. The decline in skeletal muscle mass, in these patients, leads to a rise in the specific lipid levels related to hyperlipidemia and the development of cardiometabolic syndromes. EGCG and coconut oil supplementation determined a significant decrease in CV risk, as measured by an improvement of two variables: anthropometric (such as a reduction in waist-to-hip ratio and an increase in muscle percentage) and serum analytes (with an increase in Paraoxonase 1 and albumin in the blood). Thus, the reduction in CV risk is mainly related to loss of abdominal fat, as EGCG has important anti-obesity properties by decreasing adipocyte proliferation, lipogenesis, and synthesis of inflammatory adipokines. Ketone bodies may have also contributed to this action as, in equally isoenergetic conditions, they improve basal metabolic rate and lower appetite. The positive outcome observed in patients with multiple sclerosis can be predicted also in different clinical settings, including obese subjects and patients with type 2 diabetes.

A relevant biological process that has been directly associated to the development of atherosclerotic plaque and increase risk of CVD is the process of reverse cholesterol transport (RCT). This is a physiological mechanism that protects cells from an excessive accumulation of cholesterol. Indeed, the retention of cholesterol-engorged macrophages in the arterial wall drives the formation of fatty lesions that can develop into mature atherosclerotic plaques, potentially leading to clinical CV events. Emerging experimental evidence documented that dietary fatty acids, sterols and liposoluble vitamins, may improve the RCT, thus exerting an atheroprotective action [3]. These data, although they derive from basic research in rodents and cannot be directly translated to human pathophysiology, are promising and certainly lay the bases for future research.. Future clinical studies on the atheroprotective effect of selected dietary components are mandatory in order to confirm the experimental findings.

The controversy regarding the protective/detrimental action of selected nutrients has been critically overviewed by Prof. Visioli and Dr. Poli with a focus on fatty-acid and CVD [4]. This analysis permitted them to draw some conclusions. First, saturated fatty acids are highly heterogeneous in nature and their effects most likely depend on the other food components including different classes of fatty acids they substitute. More conclusive results were obtained with monounsaturated fatty acids that, apparently, play minor roles in the CVD scenario but, when ingested via extra virgin olive oil, the presence of (poly)phenols conferred interesting cardioprotective actions. Omega 6 fatty acids, namely linoleic acid, cannot be considered harmful, and there is no reason to worry about the proportion of calories they provide within a healthy diet. Finally, the role of omega 3 fatty acids and CVD is still debated, although their intake can be considered cardioprotective via modulation of various parameters.

The last review of the Special Issue summarized the current knowledge on preclinical and clinical studies, conducted with nutraceuticals, for preventing CV calcification [5]. This analysis identified some nutraceuticals, i.e., magnesium, zinc, iron, vitamin K, and phytate, with clinical evidence on their effect on the progression of vascular calcification in high-risk population of patients. Although a lot of effort has been made on this topic, the optimal dose of nutraceuticals has not been identified and large interventional trials are warranted to support their protective effects on CV calcification.

Thus, there is no doubt that dietary factors can influence the development of CVD either directly or through their action on traditional risk factors, such as plasma lipids, blood pressure, or glucose levels. There are two main examples of dietary patterns that have been more extensively evaluated in terms of impact on CV risk, such as the DASH (Dietary Approaches to Stop Hypertension), mainly related to blood pressure control, and the Mediterranean diet. Both approaches have proved to be effective in reducing CV risk factors and potentially to prevent atherosclerotic CVD, with the Mediterranean diet emphasizing the protective effect of extra-virgin olive oil. Epidemiological studies demonstrated a reduced incidence of CVD with the Mediterranean diet that was also proved in randomized control trials to be effective in reducing CV events in primary and secondary prevention. In particular, the PREDIMED (Prevencion con Dieta Mediterranea) trial indicated that participants allocated to a Mediterranean diet, supplemented with extra-virgin olive oil or nuts, had a significantly lower (approximately 30%) incidence of major CV events compared with subjects on a low-fat diet. Nevertheless, despite the great contribution given by the results of PREDIMED trial to the available literature on this issue, randomized control trials cannot represent the sole grounds on which dietary recommendations should rely. The combination of large observational cohort studies and relatively short-term randomized trials with CV risk factors as outcomes (LDL-C and hsCRP), as reported in this Special Issue, must be considered for the determination of the impact of healthy lifestyle on CV prevention.

In conclusion, the present Special Issue effectively addressed the effects of some nutritional intervention for preventing atherosclerotic CVD or for modifying CV risk factors and the underlying mechanisms that explain their biological effects, suggesting that functional foods or nutraceuticals may thus be considered as supplement to reduce the CV risk status. Expanding our knowledge base in this area will be helpful for refining future dietary recommendations for health promotion.

## Figures and Tables

**Figure 1:**
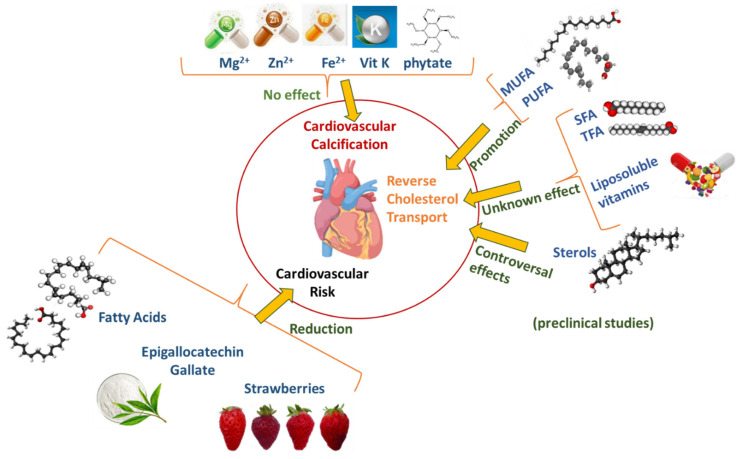
Effects of nutritional interventions on CV risk and related risk factors. Strawberry intake exerted positive cardiometabolic effect by improving insulin sensitivity in obese patients; ECGC determined a significant decrease in CV risk in patients with multiple sclerosis; emerging experimental evidence documented differential positive effects of dietary fatty acids, sterols and liposoluble vitamins on reverse cholesterol transport (RCT); supplementation with nutraceuticals, i.e., magnesium, zinc, iron, vitamin K, and phytate, has been studied for the effects on the progression of vascular calcification however no conclusive data have been obtained yet; dietary fatty acids intake had positive effects on CV risk. Abbreviations: Mg^2+^, magnesium; Zn^2+^, zinc, Fe^3+^, iron, Vit K, vitamin K; MUFA, monoinsaturated faccty acids; PUFA, polyinsaturated fatty acids; SFA, saturated fatty acids; TFA, trans fatty acids; ECGC, epigallocatechin gallate.
